# Transplantation and Employment Earnings in Kidney Transplant Recipients

**DOI:** 10.1001/jamanetworkopen.2025.60157

**Published:** 2026-02-19

**Authors:** Daisy Thomas, Calvin Diep, Ella Huszti, Juan Pablo Diaz-Martinez, Duminda Wijeysundera, Christopher D. Witiw, Blayne A. Sayed, Karim S. Ladha

**Affiliations:** 1 University of Toronto, Toronto, Ontario, Canada; 2 Department of Anesthesiology and Pain Medicine, University of Toronto, Toronto, Ontario, Canada; 3 Biostatistics Department, University Health Network, Toronto, Ontario, Canada; 4 Institute of Health Policy, Management and Evaluation, Dalla Lana School of Public Health, University of Toronto, Toronto, Ontario, Canada; 5 Department of Anesthesia, St. Michael’s Hospital, Toronto, Ontario, Canada; 6 Li Ka Shing Knowledge Institute, St. Michael’s Hospital, Toronto, Ontario, Canada; 7 Division of Neurosurgery, Department of Surgery, University of Toronto, Toronto, Ontario, Canada; 8 Ajmera Transplant Centre, HBP & Multi-Organ Transplant Program, University Health Network, Toronto, Ontario, Canada; 9 Department of Surgery, Hospital for Sick Children, Toronto, Ontario, Canada; 10 Department of Anesthesia and Pain Management, Women’s College Hospital and Toronto Western Hospital, Toronto, Ontario, Canada

## Abstract

**Importance:**

Kidney transplantation improves survival and quality of life for individuals with end-stage kidney disease (ESKD). However, its effect on objective economic outcomes, such as employment income, remains insufficiently studied.

**Objective:**

To quantify changes in employment income before and after kidney transplantation in Canada using linked administrative health and tax data.

**Design, Setting, and Participants:**

This population-based cohort study included nationwide data from Canadian adults receiving a kidney transplant between 2007 and 2016. Data were from the Canadian Hospitalization and Taxation Database (C-HAT), which includes hospital discharge records and income tax data. Eligible adults were aged 30 to 62 years; exclusions included repeat or multiorgan transplants, residents of Quebec or territories, and individuals with incomplete tax data or those in the top or bottom 1% of income earners. Data analyses were conducted between June 2024 to October 2025.

**Exposure:**

Kidney transplant.

**Main Outcome and Measures:**

The primary outcome was annual employment income, derived from tax records for 3 years before and after transplant. Employment income was defined as the combined total of employment income, self-employment income, and other employment income, and adjusted to 2023 Canadian dollars using the Bank of Canada Consumer Price Index. A linear mixed-effects model was used to evaluate changes in income over time.

**Results:**

The final cohort included 3230 individuals (mean [SD] age, 47 [8.4] years), of whom 1120 (34.7%) were female and 2630 (81.4%) were aged 30 to 55 years. Pretransplant, income declined by $4293 per year (95% CI, −$4726 to −$3861 per year; *P* < .001); posttransplant, income increased by $1006 per year (95% CI, $406 to $1605; *P* = .001).

**Conclusions and Relevance:**

In this cohort study of 3230 kidney transplant recipients, kidney transplant was associated with a reversal of declining employment income, indicating meaningful economic recovery. These findings highlight the broader socioeconomic value of transplantation and may inform policies that support patients during pretransplant vulnerability and facilitate successful return to work.

## Key Points

**Question:**

How does annual employment income change before and after kidney transplant in working-aged adults?

**Findings:**

In this cohort study of 3230 Canadian kidney transplant recipients aged 30 to 62 years, annual employment income declined by $4293 per year during the 3 years before transplant and increased by $1006 per year during the 3 years after transplant; both changes were statistically significant.

**Meaning:**

Kidney transplant was associated with a reversal of declining employment income, suggesting potential for economic recovery and the need for supportive policies before and after transplant.

## Teaser

This cohort study of Canadian adults receiving kidney transplants examines changes in annual employment income in 3-year periods prior to and following transplant.

## Introduction

End-stage kidney disease (ESKD) is characterized by the kidney’s permanent inability to filter blood and maintain internal homeostasis.^1^ The physical and mental symptoms of ESKD, alongside the demands of dialysis, create significant challenges for patients in maintaining employment, resulting in reduced productivity and income.^2,3,4^ Kidney transplantation aims to improve survival and quality of life for individuals with ESKD by restoring kidney function through donor organ replacement.^5^ One patient-centered outcome of posttransplant rehabilitation is the recipient’s ability to return to work, which has been previously correlated with enhanced mental and social well-being.^6,7,8^

While the medical benefits of kidney transplantation, such as increased lifespan, improved energy levels, and better nutritional status, are well-documented, its economic impact, has been less studied.^9^ There have only been a few studies that have explored this question, with existing research largely relying on self-reported data from surveys and qualitative interviews.^7,10,11^ These studies provide important insights but are unable to quantify changes in individual income at a national level. This population-based retrospective cohort study examined the association of kidney transplantation with employment income using data from the Canadian Hospitalization and Taxation Database (C-HAT), which combines administrative health data with income tax records.

## Methods

Research ethics board approval was not required for this study, as the data were accessed through the Statistics Canada Research Data Centre, where all analyses are conducted in accordance with Statistics Canada’s confidentiality and privacy regulations. Informed consent was not required because data were deidentified. This report follows Strengthening the Reporting of Observational Studies in Epidemiology (STROBE) reporting guidelines for cohort studies.

### Data Source

A retrospective cohort study was conducted using the Canadian Hospitalization and Taxation (C-HAT) database, developed by Statistics Canada. This database links 2 primary sources: the Discharge Abstract Database (DAD) and the T1 Family File (T1FF).^12^ The DAD, maintained by the Canadian Institute for Health Information, provides high-quality administrative data on acute care hospitalizations across Canada, excluding Quebec. The T1FF, supplied by the Canada Revenue Agency, contains detailed information on income, select demographics, and residency. At the time of analysis, data from 2004 to 2019 were available through the C-HAT database.

### Study Population

Patients aged 30 to 62 years who underwent kidney transplant between 2007 and 2016 were identified using the Canadian Classification of Health Interventions (CCI) code 1.PC.85.LA-XX, which captures recipients of either living or deceased donor kidneys.^13,14^ The age range of 30 to 62 years was selected to capture individuals likely to be stably integrated into the workforce prior to the typical Canadian retirement age 65 years. The upper age limit of 62 years ensured measurement of employment income for at least three years postprocedure.

### Exclusion Criteria

Combined organ transplants (eg, kidney-pancreas, kidney-liver) and nonindex kidney transplants (ie, repeat transplants) were excluded to maintain a focused analysis. Individuals residing in Quebec, the Northwest Territories, Yukon, Nunavut, and those with out-of-country residence were excluded. Quebec was excluded as its hospitals do not contribute to the Discharge Abstract Database (DAD), while the Northwest Territories, Yukon, and Nunavut were excluded due to lower linkage rates reported by Statistics Canada.^11^ Additionally, individuals with missing tax data 1 year prior to surgery were also removed, and the top and bottom 1% of earners were trimmed to reduce the influence of outliers.

### Outcome

To evaluate the association of surgery with employment income, annual tax records were obtained from the T1 Family File (T1FF) for individuals in the sample population. These records covered the period from three years before to three years after surgery. This time frame reflects the average 3 to 4 year waitlist duration for deceased-donor kidney recipients between 2014 and 2023, according to the Canadian Institute for Health Information (CIHI).^15^ Employment income was defined as the combined total of T4 employment income, self-employment income, and other employment income. All income values were adjusted for inflation to 2023 Canadian dollars using the Bank of Canada’s Consumer Price Index (CPI).

### Statistical Analyses

Annual employment income was modeled using a linear mixed-effects model with the formula *income ~ years × intervention + (years | STC_ID)*, where *years* represents time relative to surgery and *intervention* a binary variable indicating presurgery (0) or postsurgery (1) periods and *STC_ID* indicates a unique patient identifier. The interaction term between *years* and *intervention* models how income changes over time in association with transplant, allowing for different slopes before and after surgery. The model also includes random intercepts and slopes for years for each patient (using the unique patient identifier), accounting for individual differences in baseline income and income trajectories over time. To align patients who received transplants in the same year but different months, the binary intervention variable was set to 1 starting from the calendar year after the transplant year. This approach ensured consistent classification of presurgery and postsurgery periods. All statistical analyses were conducted in R version 4.1.1 (R Project for Statistical Computing) using the lme4, lmerTest, merTools, comorbidity, and ggplot2 packages. Data analyses were conducted between June 2024 to October 2025.

Subgroup analyses were defined to evaluate the association of age, biological sex, postoperative complications, location, transplant type, and Elixhauser Comorbidity Index Score with changes in employment income from before to after transplant. The study population was stratified by each variable of interest, and the same linear mixed-effects model was applied within each subgroup. Age was categorized as 30 to 55 years and 56 years or older to account for early retirement.^14^ Postoperative complications were studied by screening patient records for major complications using a broad range of *International Statistical Classification of Diseases and Related Health Problems, Tenth Revision (ICD-10)* and Canadian Classification of Health Interventions (CCI) codes within 30 days of the index discharge, consistent with a prior large population-based cohort study (eTable 1 in Supplement 1).^16^ Rural vs urban residence was determined using the first 3 characters of the patient’s postal code (forward sortation area), with a second character of zero indicating rural residency. Transplant type was classified using CCI procedure codes: kidney transplants from living donors (1.PC.85.LA-XX-J) and from deceased donors (1.PC.85.LA-XX-K). Finally, to understand how comorbid disease may affect this association, *ICD-10* diagnostic codes from the index surgical visit were used to calculate the Elixhauser Comorbidity Index. Participants were then grouped by score into 2 categories, 0-2 and 3-6, with the highest observed score being 6. A sensitivity analysis was performed using the same inclusion and exclusion criteria; however, the top and bottom 1% of earners were retained. Statistical significance was assessed using 2-sided tests with α = .05.

## Results

**Figure 1.**
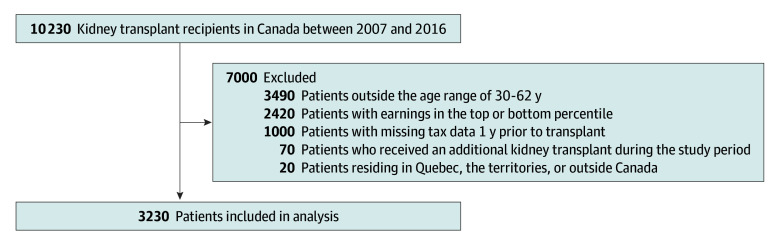
Study Sample Flow Diagram

**Figure 2.**
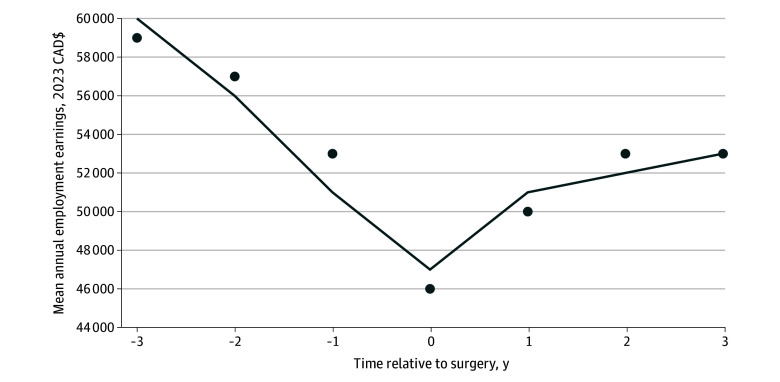
Mean Annual Employment Earnings 3 Years Before and After Kidney Transplant Annual earnings were estimated from a linear mixed-effects model for a sample of 3230 patients receiving kidney transplants. The line represents model-estimated earnings from 3 years before to 3 years after transplant. Dots represent observed mean annual earnings in the sample.

The final sample included 3230 individuals, with a mean (SD) age of 47 (8.4) years. Of these, 1120 (34.7%) were female and 2630 (81.4%) were aged 30 to 55 years (Figure 1, Table 1). The mean (SD) standardized employment income 3 years prior to transplant was CAD $59 100 (59 618) (eFigure 1 in Supplement 1). In the year of transplant, the mean (SD) employment income was $45 600 (46 833). Three years posttransplant, the mean (SD) employment income was $52 900 (53 329). Employment income was tracked annually for up to 3 years before and 3 years after kidney transplant, providing a total follow-up period of 7 years per participant. A total of 21 770 tax files were extracted, spanning the 7-year period surrounding transplant. However, 870 tax files were unavailable for certain years due to individuals not filing tax returns during this timeframe. Among the sample, annual employment income decreased $4293.30 per year (95% CI, −$4726.17 to −$3860.46; *P* < .001) prior to transplant. Following surgery, employment income increased annually by $1005.70 (95% CI, $406.09 to $1605.29; *P* = .001) (Figure 2, Table 2).

**Table 1.** Demographic and Clinical Characteristics of Cohort Who Underwent Kidney Transplant, 2007-2016

Characteristic	Patients, No. (%) (N = 3230)
Biological sex	
Female	1120 (34.7)
Male	2110 (65.3)
Complications experienced within 30 d of index discharge	
Complications	500 (15.5)
No complications	2730 (84.5)
Age, y	
Mean (SD), y	47 (8.4)
30-55	2630 (81.4)
56-62	610 (18.9)
Location	
Urban	2780 (86.1)
Rural	450 (13.9)
Donor type	
Living-donor transplant	1760 (54.5)
Deceased-donor transplant	1480 (45.8)
Elixhauser Comorbidity Index at index surgery admission	
0-2	2630 (81.4)
3-6	600 (18.6)
Participants by year of transplant^a^	
2007	350 (10.8)
2008	310 (9.6)
2009	320 (9.9)
2010	330 (10.2)
2011	300 (9.3)
2012	340 (10.5)
2013	310 (9.6)
2014	290 (9.0)
2015	330 (10.2)
2016	360 (11.1)
Annual employment income relative to kidney transplant, mean (SD), CAD$^b^	
−3	59 100 (59 618)
−2	56 900 (48 025)
−1	53 100 (45 025)
Transplant year	45 600 (46 833)
1	50 300 (49 362)
2	52 900 (51 937)
3	52 900 (53 329)

^a^ All counts are rounded to nearest 10th to satisfy Statistics Canada rules.

^b^ Income values adjusted to 2023 Canadian dollars using the Bank of Canada Consumer Price Index (CPI).

**Table 2.** Results of the Linear Mixed-Effects Model Assessing Employment Income Before and After Kidney Transplant in the Primary Cohort (n = 3230)

Parameter	Estimate, $ (95% CI)	*P* value
Intercept	46 958.8 (45 351.04 to 48 566.50)	<.001
Years	−4293.30 (−4726.17 to −3860.46)^a^	<.001
Posttransplant	2343.40 (1058.01 to 3628.82)	<.001
Years: posttransplant	5299.00 (4675.64 to 5922.43)^b^	<.001

^a^ Coefficient for years pretransplant.

^b^ Coefficient for years posttransplant = −4293.3 + 5299.0 = 1005.7 (95% CI, 406.1 to 1605.3; *P* = .001). To isolate the change in income trajectory associated with kidney transplant, the pretransplant slope (years) was subtracted from the interaction term (years: posttransplant).

Dividing the cohort into age categories, there were 2630 patients aged 30 to 55 years. This subgroup experienced an annual $3604.50 (95% CI, −$4023.90 to −$3185.21; *P* < .001) decrease in income prior to transplant, followed by annual $1667.10 (95% CI, $1081.52 to $2252.65; *P* < .001) increase posttransplant. The remaining 610 individuals were over the age of 56 years. This cohort experienced a statistically significant decrease of $7267.30 (95% CI, −$8652.72 to −$5881.81; *P* < .001) in income prior to transplant, followed by a decline of $1769.80 (95% CI, −$3692.38 to $153.39; *P* = .07) posttransplant (eFigure 2 and eTable 2 in Supplement 1).

When stratifying the cohort by sex, the sample population included 1120 women and 2110 men. Similar declines in income were seen in both groups pretransplant, followed by increases after surgery (eFigure 3 and eTable 3 in Supplement 1).

Within 30 days of index discharge, 500 patients experienced at least 1 complication, with the most common complications being surgery-related, bleeding events, mechanical ventilation, infections, and repairs of chest or abdominal musculature (eTables 1 and 4 in Supplement 1). Patients who experienced a complication saw a statistically significant decrease in income of CAD $4242.33 prior to surgery (95% CI, −$5126.92 to −$3357.86; *P* < .001), followed by increase of $1434.06 posttransplant (95% CI, $178.81 to $2689.47; *P* = .03). Similarly, the remaining 2730 patients who did not experience a complication experienced a statistically significant decrease in income of $4302.40 (95% CI, −$4788.05 to −$3816.79; *P* < .001) prior to the procedure, followed by an increase of $922.80 (95% CI, $252.49 to $1593.05; *P* = .007**)** posttransplant (eFigure 4 and eTable 5 in Supplement 1).

A total of 2780 patients resided in urban locations, experiencing a decline of income of $4186.20 (95% CI, −$4657.42 to −$3715.03; *P* < .001) pretransplant followed by an increase of $824.10 (95% CI, $176.35 to $1471.81; *P* = .01) posttransplant. Alternatively, 450 patients resided in rural locations, experiencing a decline of income of $4923.10 (95% CI, −$6010.65 to −$3835.82; *P* < .001) prior to surgery followed by an increase of $2134.20 (95% CI, $551.33 to $3717.29; *P* = .008) posttransplant (eFigure 5 and eTable 6 in Supplement 1).

Upon stratifying by donor types, 1760 patients received a living-donor transplant and 1480 received a deceased-donor transplant. Living donor recipients experienced a decline of $4984.50 (95% CI, −$5655.91 to −$4313.14; *P* < .001) of income prior to surgery followed by a $1127.00 (95% CI, $204.00 to $2050.02; *P* = .02) increase posttransplant. Deceased donor recipients experienced a decline of −$3473.10 (95% CI, −$3980.20 to −$2965.95; *P* < .001) pretransplant followed by an increase of $848.40 (95% CI, $134.40 to $1562.49; *P* = .020) posttransplant (eFigure 6 and eTable 7 in Supplement 1).

When stratifying by comorbidity index associated with codes listed at index surgery admission, 2630 patients had a score between 0 and 2, and 600 had scores between 3 and 6. Patients who had scores between 0 and 2 experienced a decline of income of $4211.10 (95% CI, −$4703.37 to −$3718.88; *P* < .001) prior to surgery followed by an increase of $1332.70 (95% CI, $653.75 to $2011.69; *P* < .001) posttransplant. While patients with scores between 3 and 6 experienced a decline of $4659.60 (95% CI, −$5533.29 to −$3785.82; *P* < .001) prior to surgery followed by a decline of $451.40 (95% CI, −$1692.59 to $789.92; *P* = .48) posttransplant (eFigure 7 and eTable 8 in Supplement 1).

Without excluding the top and bottom 1% of earners, a final sample size of 5640 individuals was achieved. In this sample, income declined by $3106.60 (95% CI, −$3536.33 to −$2676.85; *P* < .001) prior to transplant, followed by an increase of $811.20 (95% CI, $166.34 to $1456.09; *P* = .01) (eFigure 8 and eTable 9 in Supplement 1).

## Discussion

This national population-level study examining the association of kidney transplantation with recipient income identified a consistent decline in annual employment income leading up to transplant, followed by a notable rebound afterward. The findings provide evidence of the economic recovery potential following transplant and highlight demographic differences in income trajectories. Notably, individuals over the age of 56 years and patients with higher Elixhauser Comorbidity Index scores continued to experience a decline in employment income posttransplant.

The decline in pretransplant income may be explained by a number of factors. Studies on ESKD and chronic kidney disease (CKD) report low employment rates, with limited work participation primarily due to the disease burden and the demands of dialysis treatment.^17,18,19,20^ Hemodialysis, which requires patients to visit the hospital multiple times a week or several times a day, often results in difficulty maintaining full-time employment, altered job responsibilities, and increased sick leave.^21,22^ In addition to dialysis, physical factors (such as fatigue, anemia, vascular access complications, depression) and comorbidities (such as diabetes) further hinder patients ability to work, reducing both energy levels and physical capacity.^19,21,22,23,24^ Other factors, including education, employer flexibility, insurance coverage, age, race, and the use of erythropoietin have also been linked to employment outcomes in ESKD patients.^19,25^

Previous studies have shown that kidney transplant recipients experience improvements in quality of life, health, and energy levels, which can contribute to maintaining employment or returning to work.^26,27^ However, as Kirkeskov et al^17^ noted in their systematic review on the employment of kidney transplant recipients, return to work is often influenced by a combination of personal, clinical, and work-related factors. Their study identified several predictors of return to work, including employment status prior to transplant, education level, younger age, and having a living donor transplant. Conversely, adverse effects of immunosuppressive therapy, along with health limitations including fatigue, memory problems, anxiety, early retirement, and physically demanding professions, have been linked to lower self-reported work functioning and are predictors of not returning to work.^8,10,28,29^ Overall, the current analysis suggests that the positive effects on quality of life outweigh the negative sequelae of transplant which leads to a gradual increase in employment income observed after transplant (Table 2, Figure 2).

A 1996 Canadian study by Laupacis et al^10^ followed 168 kidney transplant recipients for up to 19.5 months after transplant. Health-related quality of life was assessed using hemodialysis and transplantation questionnaires, the Sickness Impact Profile, and the time trade-off technique. Participants cited health limitations, early retirement, and personal choice as reasons for not working full-time before transplant. By 6 months posttransplant, quality of life scores had improved and remained stable throughout follow-up. Notably, the proportion of employed recipients increased from 30% pretransplant to 45% 2 years posttransplant, with factors such as health limitations, early retirement, and personal choice influencing employment after receiving a transplant.^10^

Subgroup analyses revealed consistent employment patterns across biological sex and geographic location, with declines prior to transplant followed by gradual recovery afterward. A similar pattern emerged among patients who experienced complications within 30 days postindex discharge, with employment declining pretransplant and then rebounding thereafter. Previous research on the impact of postoperative complications on employment among transplant recipients has been mixed, as some studies report minimal effects while others indicate delayed return to work or reduced likelihood of resuming employment.^29,30,31,32,33^ Individuals with higher Elixhauser Comorbidity Index scores (ie, 3-6), a measure commonly used to predict in-hospital mortality and length of stay, experienced declines in employment after transplant. One potential explanation is the fact that other comorbid conditions other than kidney disease affected workforce participation; however, this requires further study to explore the underlying reasons for this finding.^34,35^

Age also influenced employment outcomes. Individuals aged 30 to 55 years had better outcomes compared with those over 56 years, who continued to show declining annual employment income. This aligns with prior research in kidney transplant recipients, where younger patients returned to work at higher rates, likely due to better job opportunities, financial incentives, and faster postoperative recovery.^36,37,38,39,40^ Older patients, in contrast, may be less likely to return to work due to higher risks of postoperative complications, multimorbidity, early retirement, or access to pensions and tax credits.^40,41,42^

Employment patterns following transplant were similar across donor types, with declines prior to surgery followed by recovery afterward. This finding contrasts with other studies which have showed that recipients of living donor kidneys generally experience better outcomes overall, including improved health-related quality of life, greater societal participation, higher kidney function, lower risk of graft failure, and longer graft survival.^41,43,44,45^

These findings highlight the substantial economic benefits of kidney transplantation, particularly for working-age individuals who are more likely to return to the workforce and contribute to the tax base. This suggests that the long-term economic value of kidney transplantation may exceed the direct health care costs of providing the procedure. Future research should further investigate factors associated with employment outcomes among kidney transplant recipients. Clinical variables such as the etiology of ESKD and the duration of pretransplant hemodialysis, which has been linked to reduced employment, should also be explored.^16,36^ Additionally, incorporating socioeconomic factors such as family size, spousal income, and education, potentially through survey-based studies, may provide a more comprehensive understanding of the broader financial and social impacts experienced by transplant recipients. Future studies could also include a control cohort to enable a more robust assessment of age-related differences in employment outcomes.

### Strengths and Limitations

The key strength of the study is the use of objective metrics sourced from income tax records. This population-based data allowed for the assessment of employment income with a high degree of accuracy and standardization across a large cohort, offering valuable insights into posttransplant outcomes.

There are several limitations to consider when interpreting the results. First, Canadian data may not be generalizable to other jurisdictions. Additionally, the reliance on administrative health and tax data limits the ability to accurately capture patient socioeconomic factors such as education level and occupation title, which could influence employment outcomes. Furthermore, some patients lacked a complete tax record for the study period, potentially introducing gaps in income data and limiting the comprehensiveness of the analysis. The study did not account for distinctions between ESKD, chronic kidney disease (CKD), or preemptive kidney transplant, or for dialysis modality and frequency as some patients may have received diagnoses or dialysis outside hospital settings that were not captured in the DAD. Patients undergoing more intensive dialysis regimens or with greater comorbidity burden may experience more severe limitations in work capacity, potentially affecting both pre- and posttransplant income. Although efforts were made to identify and account for various postoperative complications, long-term health issues that could affect posttransplant employment income, such as diabetes, may not have been fully captured. Finally, individual wait-list times could not be determined in this study, which is an important factor as previous research has highlighted the detrimental effects of prolonged wait times, including increased risk of death, transplant failure, and reduced quality of life.^46,47,48^

## Conclusions

In this cohort study of 3230 kidney transplant recipients, kidney transplantation was associated with a reversal of declining employment income following a period of decline prior to the procedure. Most notably, younger patients demonstrated greater improvements in income after transplant compared with older recipients. Overall, the results from this study aim to inform health care stakeholders, employers, and policymakers in designing programs and policy interventions that support transplant recipients and promote their successful return to employment.

## Notes

Supplement 1. eFigure 1. Histogram of annual employment income 3 years prior to kidney transplantation (year = –3) in a sample of 3230 patients eFigure 2. Mean annual employment earnings of kidney transplant recipients by age eFigure 3. Mean annual employment earnings of kidney transplant recipients divided by sex eFigure 4. Mean annual employment earnings of kidney transplant recipients with and without complications 30 days post-transplantation eFigure 5. Mean annual employment earnings of kidney transplant recipients divided by location eFigure 6. Mean annual employment earnings of kidney transplant recipients divided by donor type eFigure 7. Mean annual employment earnings of kidney transplant recipients divided by Elixhauser Comorbidity Index eFigure 8. Mean annual employment earnings of kidney transplant recipients without excluding for top/bottom 1 percentile eTable 1. CCI and *ICD* codes used to identify complications post 30 days transplantation eTable 2. Linear mixed-effects model output analyzing employment income of kidney transplant patients stratified by age eTable 3. Linear mixed-effects model output analyzing employment income of kidney transplant patients stratified by sex eTable 4. Top 5 complications by incidence experienced by kidney transplant patients within 30-days post-discharge eTable 5. Linear mixed-effects model output analyzing employment income of kidney transplant recipients stratified by presence or absence of complications within 30 days post-discharge eTable 6. Linear mixed-effects model output analyzing employment income of kidney transplant recipients stratified by urban or rural residence eTable 7. Linear mixed-effects model output analyzing employment income of kidney transplant recipients stratified by living or deceased donor transplants eTable 8. Linear mixed-effects model output analyzing employment income of kidney transplant recipients stratified by Elixhauser Comorbidity Index eTable 9. Linear mixed-effects model output analyzing employment income of kidney transplant recipients without excluding top and bottom 1 percentile of earners (n = 5640)

Supplement 2. Data Sharing Statement
