# Supplementary material for: Transplantation and Employment Earnings in Kidney Transplant Recipients
Source: JAMA Netw Open. 2026 Feb 19;9(2):e2560157. doi: 10.1001/jamanetworkopen.2025.60157 (PMC12921529; doi:10.1001/jamanetworkopen.2025.60157)
Supplement: Supplement 1. — eFigure 1. Histogram of annual employment income 3 years prior to kidney transplantation (year = –3) in a sample of 3230 patients eFigure 2. Mean annual employment earnings of kidney transplant recipients by age eFigure 3. Mean annual employment earnings of kidney transplant recipients divided by sex eFigure 4. Mean annual employment earnings of kidney transplant recipients with and without complications 30 days post-transplantation eFigure 5. Mean annual employment earnings of kidney transplant recipients divided by location eFigure 6. Mean annual employment earnings of kidney transplant recipients divided by donor type eFigure 7. Mean annual employment earnings of kidney transplant recipients divided by Elixhauser Comorbidity Index eFigure 8. Mean annual employment earnings of kidney transplant recipients without excluding for top/bottom 1 percentile eTable 1. CCI and ICD codes used to identify complications post 30 days transplantation eTable 2. Linear mixed-effects model output analyzing employment income of kidney transplant patients stratified by age eTable 3. Linear mixed-effects model output analyzing employment income of kidney transplant patients stratified by sex eTable 4. Top 5 complications by incidence experienced by kidney transplant patients within 30-days post-discharge eTable 5. Linear mixed-effects model output analyzing employment income of kidney transplant recipients stratified by presence or absence of complications within 30 days post-discharge eTable 6. Linear mixed-effects model output analyzing employment income of kidney transplant recipients stratified by urban or rural residence eTable 7. Linear mixed-effects model output analyzing employment income of kidney transplant recipients stratified by living or deceased donor transplants eTable 8. Linear mixed-effects model output analyzing employment income of kidney transplant recipients stratified by Elixhauser Comorbidity Index eTable 9. Linear mixed-effects model output analyzing employme [file jamanetwopen-e2560157-s001.pdf]

Supplemental Online Content

Thomas D, Diep C, Huszti E, et al. Transplantation and Employment Earnings in Kidney Transplant Recipients. *JAMA Netw Open.* 2026;9(2):e2560157. doi:10.1001/jamanetworkopen.2025.60157

eFigure 1. Histogram of annual employment income 3 years prior to kidney transplantation (year = –3) in a sample of 3230 patients

eFigure 2. Mean annual employment earnings of kidney transplant recipients by age

eFigure 3. Mean annual employment earnings of kidney transplant recipients divided by sex

eFigure 4. Mean annual employment earnings of kidney transplant recipients with and without complications 30 days post-transplantation

eFigure 5. Mean annual employment earnings of kidney transplant recipients divided by location

eFigure 6. Mean annual employment earnings of kidney transplant recipients divided by donor type

eFigure 7. Mean annual employment earnings of kidney transplant recipients divided by Elixhauser Comorbidity Index

eFigure 8. Mean annual employment earnings of kidney transplant recipients without excluding for top/bottom 1 percentile

eTable 1. CCI and *ICD* codes used to identify complications post 30 days transplantation

eTable 2. Linear mixed-effects model output analyzing employment income of kidney transplant patients stratified by age

eTable 3. Linear mixed-effects model output analyzing employment income of kidney transplant patients stratified by sex

eTable 4. Top 5 complications by incidence experienced by kidney transplant patients within 30-days post-discharge

eTable 5. Linear mixed-effects model output analyzing employment income of kidney transplant recipients stratified by presence or absence of complications within 30 days post-discharge

© 2026 Thomas D et al. *JAMA Network Open*.

eTable 6. Linear mixed-effects model output analyzing employment income of kidney transplant recipients stratified by urban or rural residence

eTable 7. Linear mixed-effects model output analyzing employment income of kidney transplant recipients stratified by living or deceased donor transplants

eTable 8. Linear mixed-effects model output analyzing employment income of kidney transplant recipients stratified by Elixhauser Comorbidity Index

eTable 9. Linear mixed-effects model output analyzing employment income of kidney transplant recipients without excluding top and bottom 1 percentile of earners (n = 5640)

This supplemental material has been provided by the authors to give readers additional information about their work.

© 2026 Thomas D et al. *JAMA Network Open*.

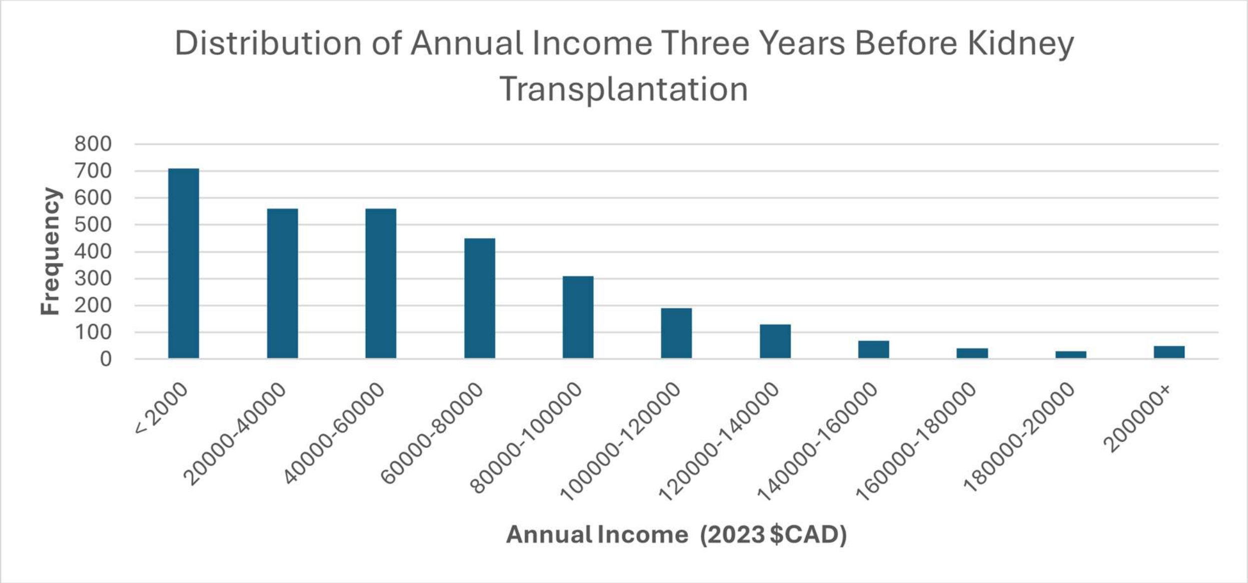

eFigure 1. Histogram of annual employment income 3 years prior to kidney transplantation (year = –3) in a sample of 3,230 patients.

© 2026 Thomas D et al. *JAMA Network Open*.

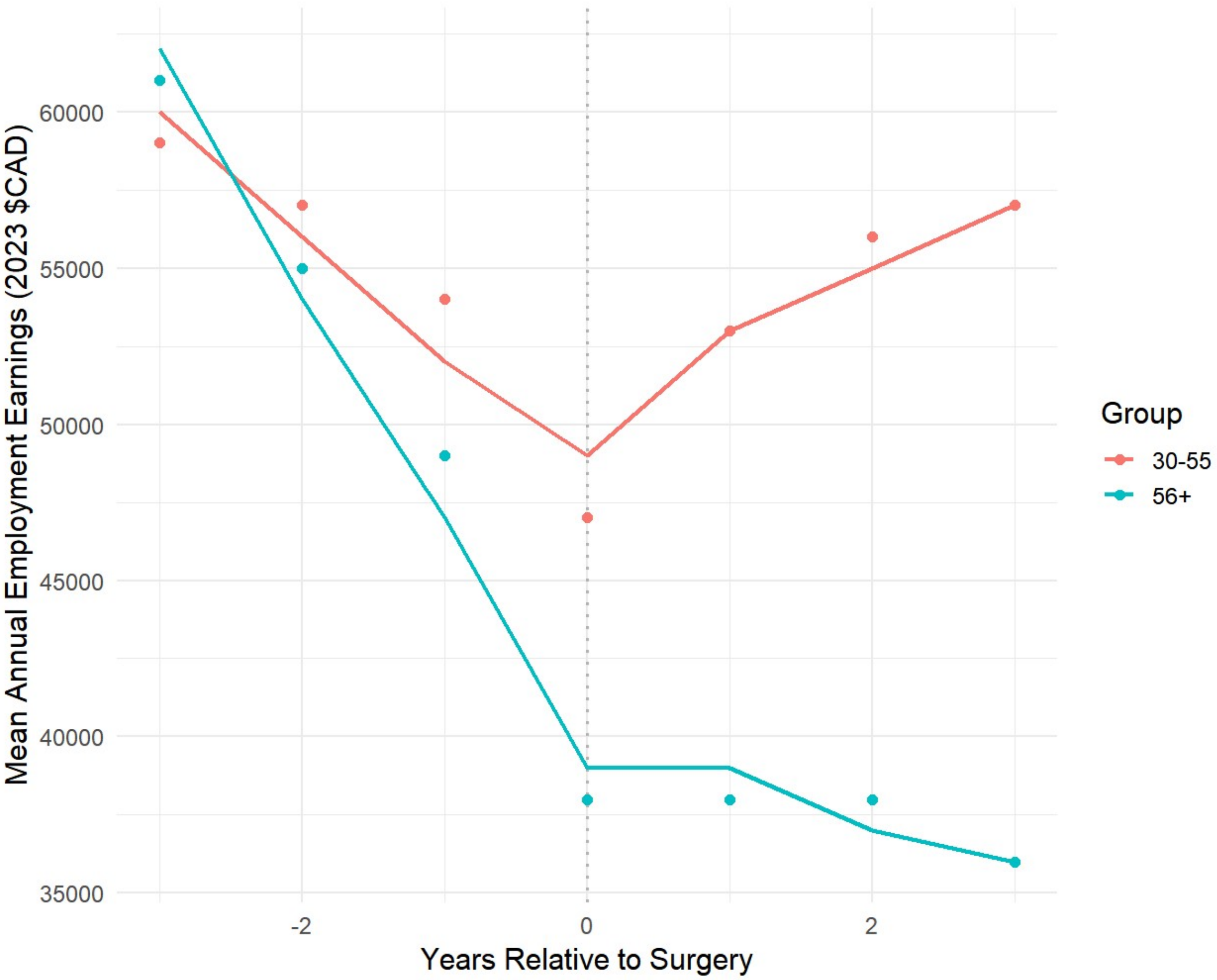

eFigure 2. Mean Annual Employment Earnings of Kidney Transplant Recipients by Age. A linear mixed-effects model, accounting for both fixed and random effects, was applied separately to subgroups of individuals aged 30-55 (n =2,630) and over 56 (n=610). The red line represents predicted employment income for individuals aged 30-55, while the blue line shows predictions for recipients over 56. Dots represent the mean annual earnings for each subgroup.

© 2026 Thomas D et al. *JAMA Network Open*.

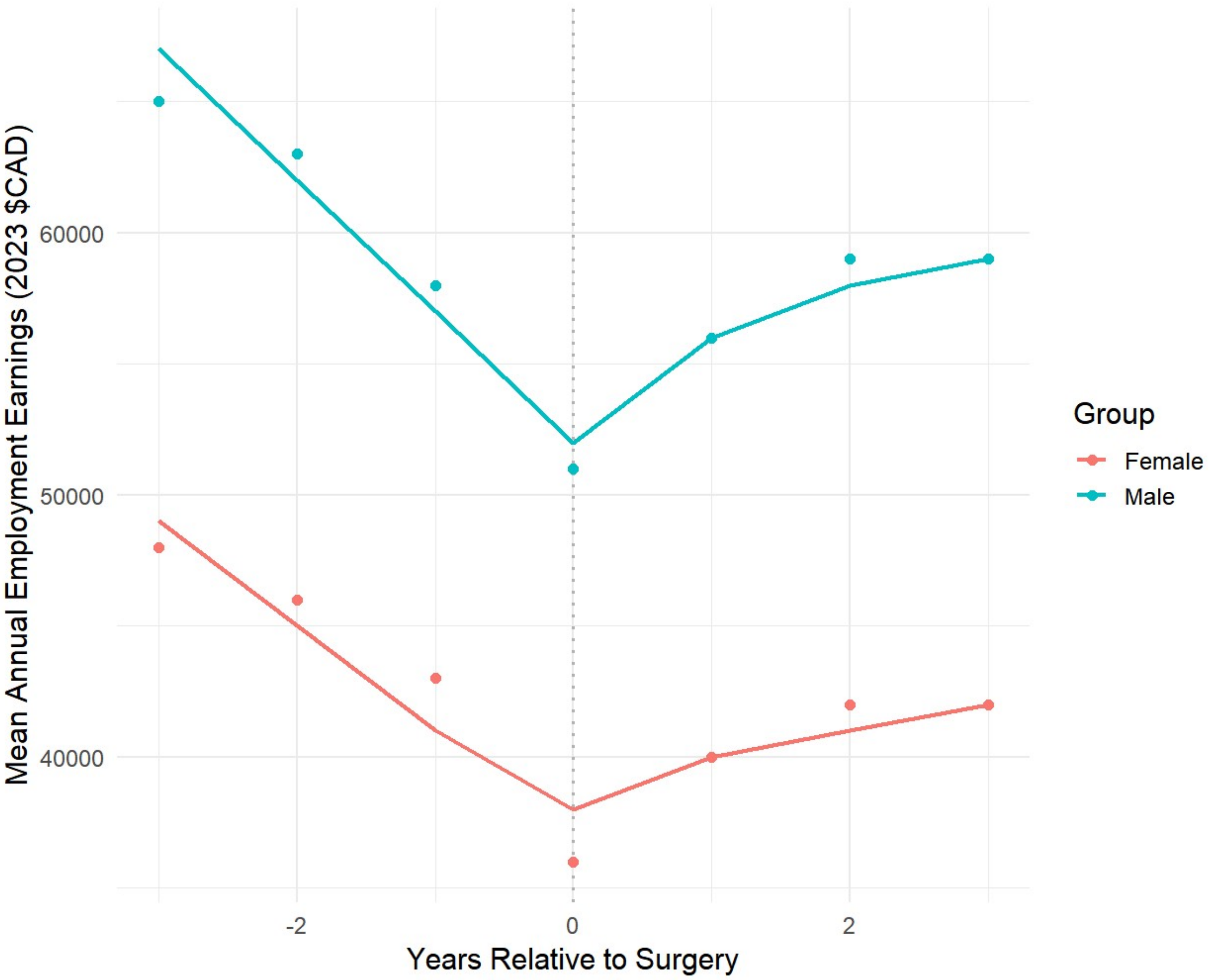

eFigure 3. Mean Annual Employment Earnings of Kidney Transplant Recipients Divided by Sex. A linear mixed-effects model, accounting for both fixed and random effects, was applied separately to male (n=2110) and female (n=1120) subgroups. The blue line represents predicted employment income for male recipients, while the red line shows predictions for female recipient. Dots represent the mean annual earnings for each subgroup.

© 2026 Thomas D et al. *JAMA Network Open*.

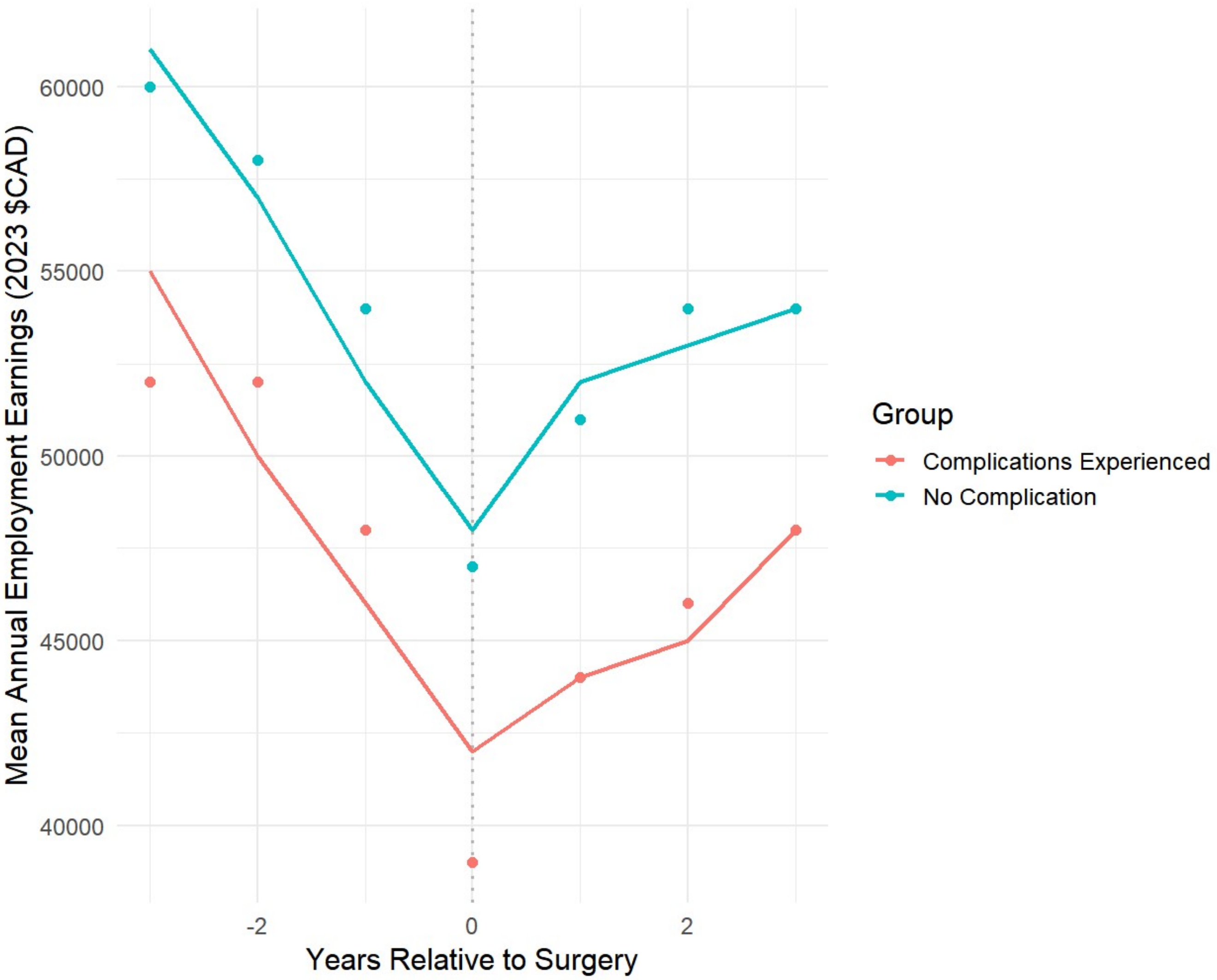

eFigure 4. Mean Annual Employment Earnings of Kidney Transplant Recipients With and Without Complications 30 Days Post-Transplantation. A linear mixed-effects model, accounting for both fixed and random effects, was applied separately to subgroups of patients with (n=500) and without (n=2,730) complications. The blue line represents predicted employment income for patients without complications, while the red line shows predictions for those with complications. Dots represent the mean annual earnings for each subgroup.

© 2026 Thomas D et al. *JAMA Network Open*.

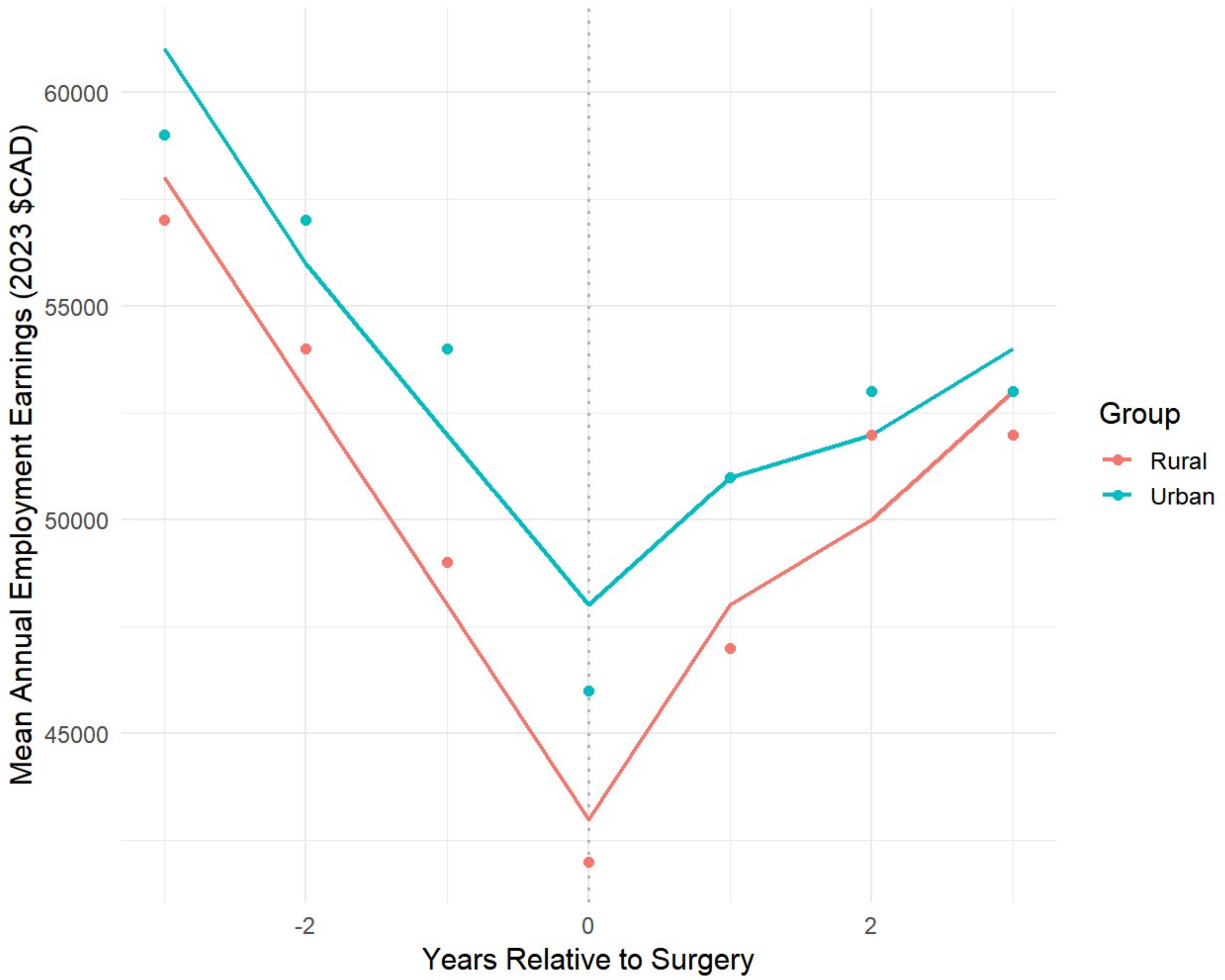

eFigure 5. Mean Annual Employment Earnings of Kidney Transplant Recipients Divided by Location. A linear mixed-effects model, accounting for both fixed and random effects, was applied separately to urban (n =2780) and rural patients (n=450). The blue line represents predicted employment income for urban recipients, while the red line shows predictions for rural recipient. Dots represent the mean annual earnings for each subgroup.

© 2026 Thomas D et al. *JAMA Network Open*.

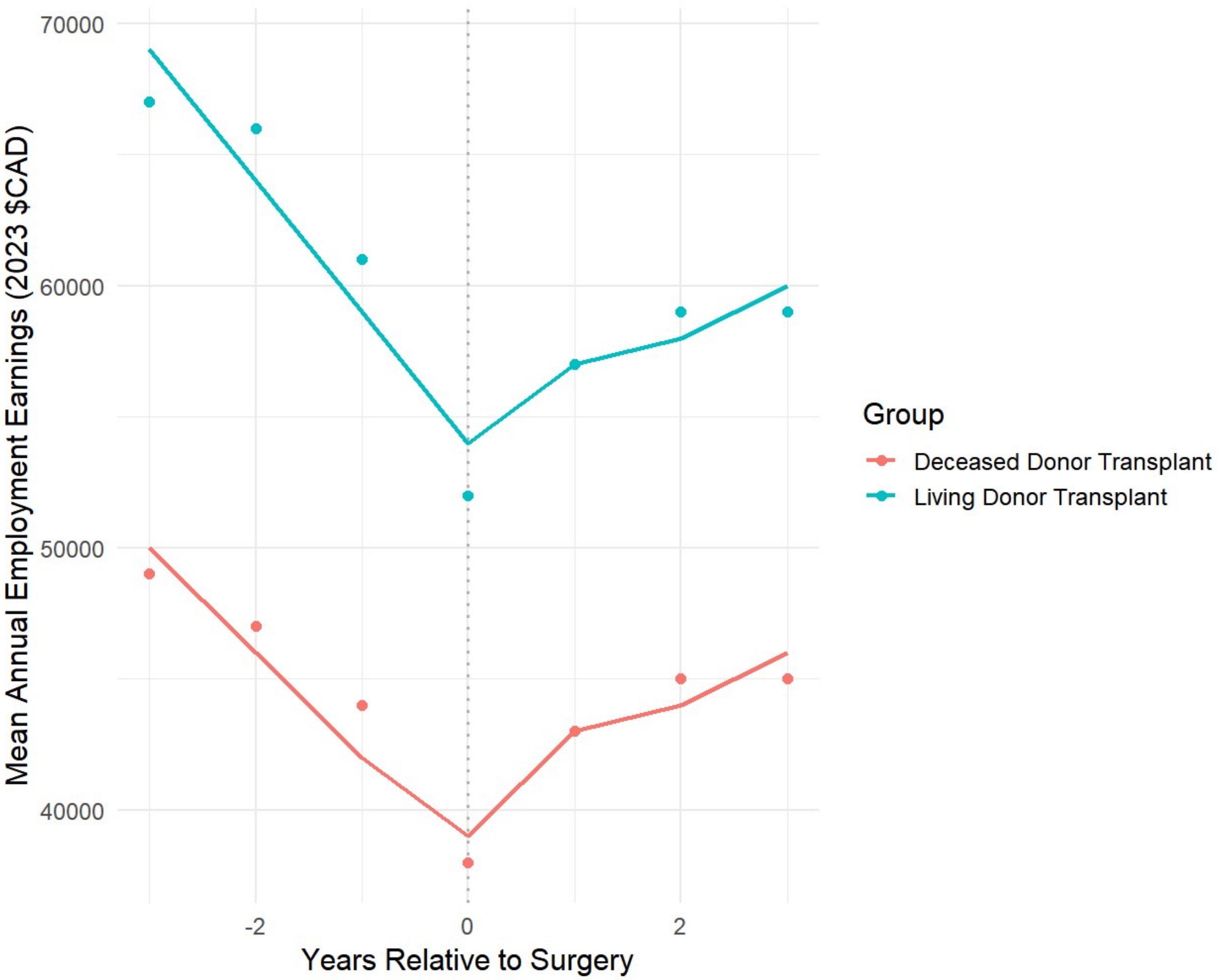

eFigure 6. Mean Annual Employment Earnings of Kidney Transplant Recipients Divided by Donor type. A linear mixed-effects model, accounting for both fixed and random effects, was applied separately to living donor (n=1760) and deceased donor (n=1480) subgroups. The blue line represents predicted employment income for living donor transplant recipients, while the red line shows predictions for deceased donor transplant recipient. Dots represent the mean annual earnings for each subgroup.

© 2026 Thomas D et al. *JAMA Network Open*.

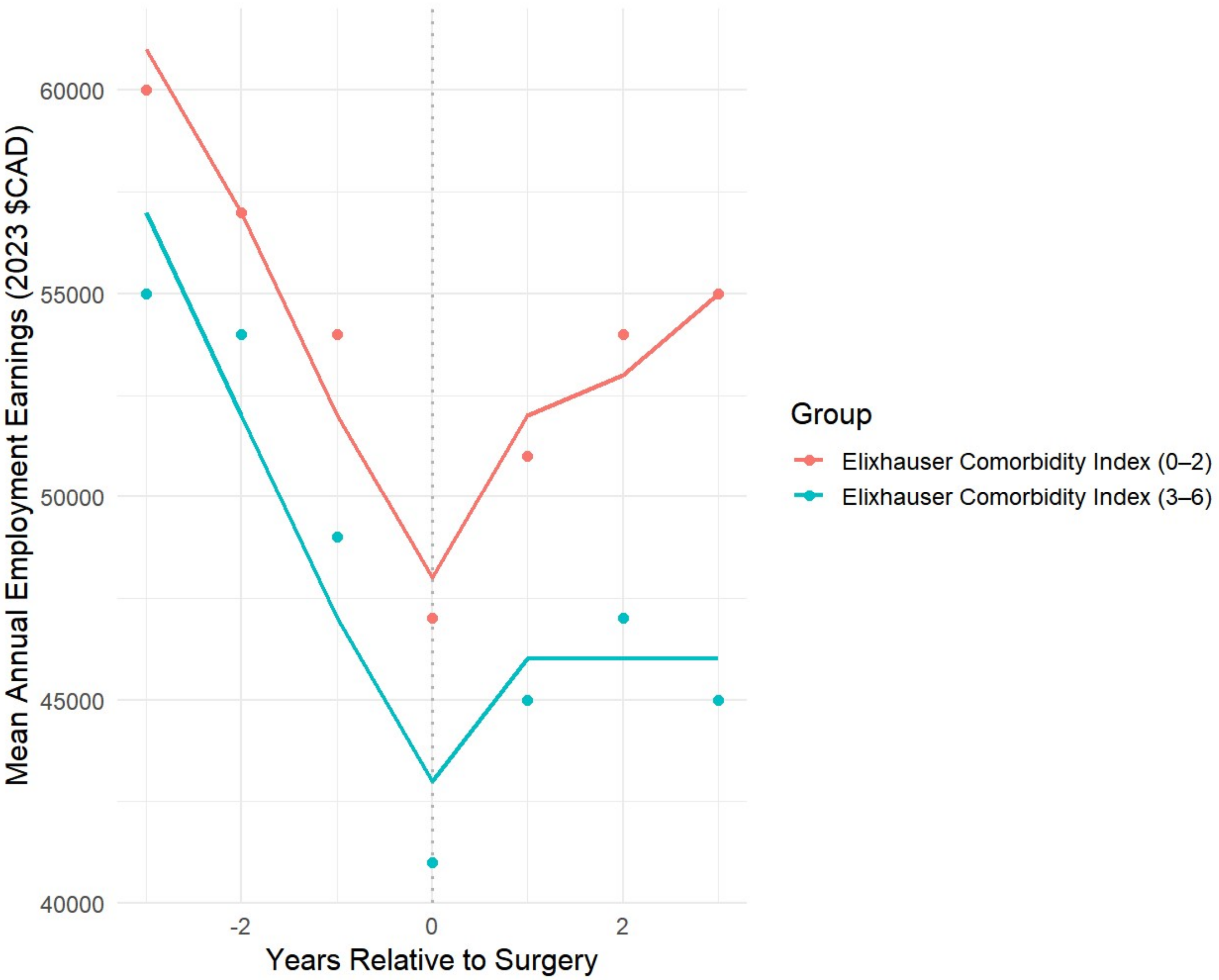

eFigure 7. Mean Annual Employment Earnings of Kidney Transplant Recipients Divided by Elixhauser Comorbidity index. A linear mixed-effects model, accounting for both fixed and random effects, was applied separately to patients with scores of 0-2 (n=2630) and 3-6 (n = 600). The blue line represents predicted employment income for male recipients, while the red line shows predictions for female recipient. Dots represent the mean annual earnings for each subgroup.

© 2026 Thomas D et al. *JAMA Network Open*.

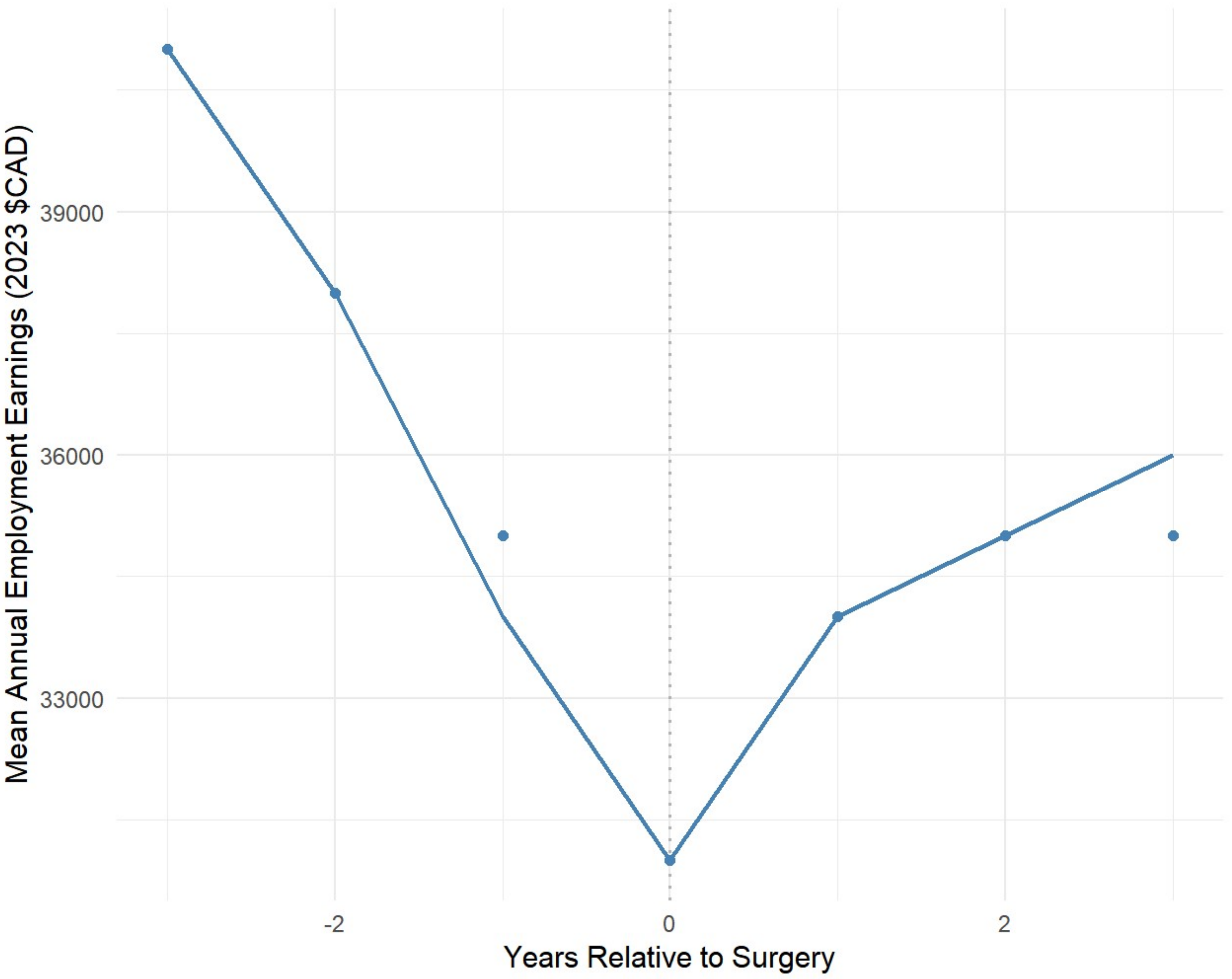

eFigure 8. Mean Annual Employment Earnings of Kidney Transplant Recipients without excluding for top/bottom 1 percentile (n = 5640) . The line represents predicted employment income for recipients, while the dots represent the mean annual earnings.

© 2026 Thomas D et al. *JAMA Network Open*.

eTable 1. CCI and ICD Codes used to identify complications post 30 days transplantation.

International Classification of Diseases (ICD) Codes	
**Description**	**Codes**
Anesthesia-related complications	Y48: Adverse effects of anaesthetics, therapeutic gases<br>T88: Other complications of surgical and medical care, not elsewhere classified
Surgery-related complications	Y60: Unintentional cut, puncture, perforation or haemorrhage during surgical and medical care<br>T81: Complications of procedures, not elsewhere classified
Bleeding	T810: Haemorrhage and haematoma complicating a procedure, not elsewhere classified<br>R58: Hemorrhage, not elsewhere classified
Delirium	F05: Delirium due to known physiological condition
Coma	R4020: Coma<br>R4029: Coma, unspecified
Cardiac arrest	I46: Cardiac arrest
Myocardial infarction	I21: Acute myocardial infarction<br>I22: Subsequent myocardial infarction<br>I23: Certain complications following acute myocardial infarction
Atrial fibrillation	I48: Atrial fibrillation and flutter
Heart failure	I50: Heart failure
Pneumonia	J12: Viral pneumonia, not elsewhere classified<br>J13: Pneumonia due to Streptococcus pneumoniae<br>J14: Pneumonia due to Haemophilus influenzae<br>J15: Bacterial pneumonia, not elsewhere classified<br>J16: Pneumonia due to other infectious organisms, not elsewhere classified<br>J17: Pneumonia in diseases classified elsewhere<br>J18: Pneumonia, unspecified organism<br>J69: Pneumonitis due to solids and liquids
Pneumothorax	J93: Pneumothorax and air leak
Pulmonary edema	J81: Pulmonary edema
Respiratory failure	R09.2: Respiratory arrest<br>J80: Acute respiratory distress syndrome<br>J96: Respiratory failure, not elsewhere classified
Pulmonary embolism	I26: Pulmonary embolism<br>T80.0: Air embolism following infusion, transfusion or therapeutic injection
Deep venous thrombosis	I80: Phlebitis and thrombophlebitis
Stroke	H34: Central retinal artery occlusion<br>I60: Nontraumatic subarachnoid hemorrhage<br>I61: Nontraumatic intracerebral hemorrhage<br>I63: Cerebral infarction<br>I64: Stroke, not specified as haemorrhage or infarction<br>G45: Transient cerebral ischemic attacks and related
Infection	T81.3: Disruption of operation wound, not elsewhere classified<br>T81.4: Infection following a procedure, not elsewhere classified
Sepsis	A40: Streptococcal sepsis<br>A41: Other sepsis<br>R65: Systemic Inflammatory Response Syndrome
Shock	R57: Shock, not elsewhere classified
**Canadian Classification of Health Interventions (CCI) codes**	
Bleeding requiring transfusion	1.LZ.19
Mechanical ventilation	1.GZ.31
Repair of the chest and abdominal muscles - hernia repair	1.SY.80

© 2026 Thomas D et al. *JAMA Network Open*.

eTable 2. Linear Mixed-Effects Model Output Analyzing Employment Income of Kidney Transplant Patients Stratified by Age

	Parameter	Estimate (95% CI)	p-value
Ages 30-55 (n = 2630)	**Intercept**	48663.2 (46,886.5 - 50,439.9)	P < .001
	**Years** [a]	-3604.5 (-4,023.9 to -3,185.2)	P < .001
	**Intervention**	2703.4 (1,432.9 - 3,973.9)	P < .001
	**Years: Intervention** [b]	5271.6 (4,655.6 - 5,887.7)	P < .001
Ages 56-62 (n = 610)	**Intercept**	39555.4 (35,843.2 - 43,267.7)	P < .001
	**Years** [c]	-7267.3 (-8,652.7 to -5,881.8)	P < .001
	**Intervention**	740.6 (-3,342.7 to 4,823.5)	P = .72
	**Years: Intervention** [d]	5497.5 (3,518.6 - 7,477.4)	P < .001

To isolate the change in income trajectory caused by kidney transplantation, the pre-transplant slope (Years) was subtracted from the interaction term (Years: Post-transplantation).

[a] **Coefficient for Years pre-transplant,** [b] **Coefficient for years post-transplant** = -3604.5 +5271.6 = 1667.1 (95% CI, 1081.5 - 2252.7; P <.001)

[c] **Coefficient for Years pre-transplant,** [d] **Coefficient for years post-transplant** = -7267.3 + 5497.5 = -1769.8 (95% CI, -3692.4 to 153.4; P=.071)

© 2026 Thomas D et al. *JAMA Network Open*.

eTable 3. Linear Mixed-Effects Model Output Analyzing Employment Income of Kidney Transplant Patients Stratified by sex

	Parameter	Estimate (95% CI)	p-value
Female (n = 1120)	**Intercept**	37,756.4 (35,547.1 - 39,965.7)	P < .001
	**Years[a]**	-3,604.5 (-4,161.0 to -3,047.9)	P < .001
	**Intervention**	1,146.2 (-494.8 to 2,787.3)	P = .17
	**Years: Intervention [b]**	4,597.8 (3,804.1 - 5,391.4)	P < .001
Male (n = 2110)	**Intercept**	51,851.9 (49,723.3 - 53,980.4)	P < .001
	**Years[c]**	-4,667.2 (-5,261.3 to -4,073.2)	P < .001
	**Intervention**	2979.1 (1,211.5 - 4,747.1)	P < .001
	**Years: Intervention [d]**	5686.1 (4,827.4 - 6,545.0)	P < .001

To isolate the change in income trajectory caused by kidney transplantation, the pre-transplant slope (Years) was subtracted from the interaction term (Years: Post-transplantation).

[a] **Coefficient for Years pre-transplant,** [b] **Coefficient for years post-transplant** = -3,604.5 + 4,597.8 = $993.3 (95% CI, 226.8 - 1759.7; P= .011)

[c] **Coefficient for Years pre-transplant,** [d] **Coefficient for years post-transplant** = -4,667.2 +5686.1 = 1018.9 (95% CI, 193.9 - 1843.8; P=.015)

© 2026 Thomas D et al. *JAMA Network Open*.

eTable 4. Top Five Complications by Incidence experienced by Kidney Transplant Patients within 30-days post-discharge

Complication	Count
Surgery-related complications	290
Bleeding events	190
Mechanical ventilation	160
Infections	60
Repairs of chest or abdominal musculature	50
Others	180
**Total**	930

© 2026 Thomas D et al. *JAMA Network Open*.

eTable 5. Linear Mixed-Effects Model Output Analyzing Employment Income of Kidney Transplant Recipients Stratified by Presence or Absence of Complications Within 30 Days Post-Discharge

	Parameter	Estimate (95% CI)	p-value
Complications 30 days post-operation (n =500)	**Intercept**	41156.3 (37,468.5 - 44,843.8)	P < .001
	**Years[a]**	-4242.3 (-5,126.9 to -3,357.9)	P < .001
	**Intervention**	32.3 (-2,676.3 to 2,741.2)	P = .98
	**Years: Intervention [b]**	5676.4 (4,357.2 - 6,995.8)	P < .001
No Complications (n = 2730)	**Intercept**	48029.5 (46,253.9 - 49,805.0)	P < .001
	**Years[c]**	-4302.4 (-4,788.1 to -3,816.8)	P < .001
	**Intervention**	2758.8 (1,322.5 - 4,195.0)	P < .001
	**Years: Intervention [d]**	5225.2 (4,529.2 - 5,921.3)	P < .001

To isolate the change in income trajectory caused by kidney transplantation, the pre-transplant slope (Years) was subtracted from the interaction term (Years: Post-transplantation).

[a] **Coefficient for Years pre-transplant,** [b] **Coefficient for years post-transplant** =-4242.3+5676.4 = $1,434.1 (95% CI, 178.8 - 2689.5, P= .025).

[c] **Coefficient for Years pre-transplant,** [d] **Coefficient for years post-transplant** =-4302.4 + 5225.2= $922.8 (95% CI, 252.5 - 1593.1; P = .007)

© 2026 Thomas D et al. *JAMA Network Open*.

eTable 6. Linear Mixed-Effects Model Output Analyzing Employment Income of Kidney Transplant Recipients Stratified by Urban or Rural Residence

	Parameter	Estimate (95% CI)	p-value
Urban (n = 2780)	**Intercept**	47635 (45890.4 - 49379.6)	P < .001
	**Years[a]**	-4186.2 (-4657.4 to -3715)	P < .001
	**Intervention**	2295.9 (916.2 - 3675.7)	P = .001
	**Years: Intervention [b]**	5010.3 (4341.3 - 5679.4)	P < .001
Rural (n = 450)	**Intercept**	42839.8 (38712.9 - 46966.6)	P < .001
	**Years[c]**	-4923.1 (-6010.6 to -3835.8)	P < .001
	**Intervention**	2561.9 (-963.4 to 6086.2)	P = .15
	**Years: Intervention [d]**	7057.4 (5346.7 to 8768.3)	P < .001

To isolate the change in income trajectory caused by kidney transplantation, the pre-transplant slope (Years) was subtracted from the interaction term (Years: Post-transplantation).

[a] **Coefficient for Years pre-transplant,** [b] **Coefficient for years post-transplant** = -4186.2 + 5010.3 = 824.1 (P = .013, 95% CI: 176.346 - 1471.8).

[c] **Coefficient for Years pre-transplant,** [d] **Coefficient for years post-transplant** = -4923.1 + 7057.4 = 2134.3 (P =.008, 95% CI: 551.3 to 3717.3).

© 2026 Thomas D et al. *JAMA Network Open*.

eTable 7. Linear Mixed-Effects Model Output Analyzing Employment Income of Kidney Transplant Recipients Stratified by Living or Deceased Donor Transplants

	Parameter	Estimate (95% CI)	p-value
Living Donor Transplant (n = 1760)	**Intercept**	53645.5 (51284.7 - 56006.4)	P < .001
	**Years[a]**	-4984.5 (-5655.9 to -4313.1)	P < .001
	**Intervention**	1900.8 (-67.2 to 3868.7)	P = .058
	**Years: Intervention [b]**	6111.5 (5157.4 - 7065.9)	P < .001
Deceased Donor Transplant (n = 1480)	**Intercept**	38985.2 (36947.4 - 41022.9)	P < .001
	**Years[c]**	-3473.1 (-3980.2 to -2966)	P < .001
	**Intervention**	2878.8 (1326.2 - 4431.3)	P < .001
	**Years: Intervention [d]**	4321.5 (3568.613 - 5074.569	P < .001

To isolate the change in income trajectory caused by kidney transplantation, the pre-transplant slope (Years) was subtracted from the interaction term (Years: Post-transplantation).

[a] **Coefficient for Years pre-transplant,** [b] **Coefficient for years post-transplant** = -4984.5 + 6111.5 = 1127 (95% CI, 203.9 - 2050; P = 0.017)

[c] **Coefficient for Years pre-transplant,** [d] **Coefficient for years post-transplant** = -3473.1 + 4321.5 = 848.4 (95% CI, 134.4 to 1562.5; P = .020)

© 2026 Thomas D et al. *JAMA Network Open*.

eTable 8. Linear Mixed-Effects Model Output Analyzing Employment Income of Kidney Transplant Recipients Stratified by Elixhauser Comorbidity Index

	Parameter	Estimate (95% CI)	p-value
Score between 0-2 (n = 2630)	**Intercept**	47929.4 (46127.2 - 49731.6)	P < .001
	**Years[a]**	-4211.1 (-4703.4 to -3718.9)	P < .001
	**Intervention**	2103.1 (652.7 - 3553.4)	P = .004
	**Years: Intervention [b]**	5543.8 (4840.2 to 6247.5)	P < .001
Score between 3-6 (n = 600)	**Intercept**	42665.7 (39154.4 - 46177)	P < .001
	**Years[c]**	-4659.6 (-5533.3 to -3785.8)	P < .001
	**Intervention**	3433.8 (713.3 - 6154.3)	P = .01
	**Years: Intervention [d]**	4208.2 (2891.4 - 5525.5)	P < .001

To isolate the change in income trajectory caused by kidney transplantation, the pre-transplant slope (Years) was subtracted from the interaction term (Years: Post-transplantation).

[a] **Coefficient for Years pre-transplant,** [b] **Coefficient for years post-transplant** = -4211.1 +5543.8 = 1,332.7 (95% CI, 653.7 - 2011.7; P < .001).

[c] **Coefficient for Years pre-transplant,** [d] **Coefficient for years post-transplant** = -4659.6 + 4208.2 = -451.4 (95% CI, -1692.6 to 789.9; P =.48)

© 2026 Thomas D et al. *JAMA Network Open*.

eTable 9. Linear Mixed-Effects Model Output Analyzing Employment Income of Kidney Transplant Recipients without excluding top/bottom 1 percentile of earners (n = 5640)

Parameter	Estimate (95% CI)	p-value
**Intercept**	31102.5<br>(29477.5 - 32727.6)	P < .001
**Years**[c]	-3106.6<br>(-3536.3 to -2676.9)	P < .001
**Intervention**	1594.0<br>(103.7 - 3084.3)	P = .036
**Years: Intervention** [d]	3917.8<br>(3195.7 - 4640.0)	P < .001

To isolate the change in income trajectory caused by kidney transplantation, the pre-transplant slope (Years) was subtracted from the interaction term (Years: Post-transplantation).

[a] **Coefficient for Years pre-transplant,** [b] **Coefficient for years post-transplant** =-3106.6 + 3917.8 = 811.2 (95% CI, 166.3 to 1456.1, P = 0.014).

© 2026 Thomas D et al. *JAMA Network Open*.
