# Supplementary material for: Transplantation and Employment Earnings in Kidney Transplant Recipients
Source: JAMA Netw Open. 2026 Feb 19;9(2):e2560157. doi: 10.1001/jamanetworkopen.2025.60157 (PMC12921529; doi:10.1001/jamanetworkopen.2025.60157)
Supplement: Supplement 2. — Data Sharing Statement [file jamanetwopen-e2560157-s002.pdf]

Data Sharing Statement

Thomas. Transplantation and Employment Earnings in Kidney Transplant Recipients. *JAMA Netw Open*. Published February 19, 2026. doi:10.1001/jamanetworkopen.2025.60157

Data
Data available: No

Additional Information
Explanation for why data not available: As Statistics Canada is responsible for safeguarding the data used in this research, the authors are unable to share the data directly. Data access requests and inquiries can be made directly with Statistics Canada, which manages all data sharing and usage restrictions.
